# Effects of Simulated Solar Wind on Polymethyl Methacrylate Thin Film

**DOI:** 10.3390/nano12121992

**Published:** 2022-06-10

**Authors:** Lidia Mezzina, Angelo Nicosia, Giuseppe Antonio Baratta, Maria Elisabetta Palumbo, Carlotta Scirè, Placido Giuseppe Mineo

**Affiliations:** 1 Department of Chemical Sciences and INSTM UdR of Catania, University of Catania, V.le A. Doria 6, 95125 Catania, Italy; lidia.mezzina@phd.unict.it (L.M.); angelo.nicosia@unict.it (A.N.); 2 INAF—Osservatorio Astrofisico di Catania, Via S. Sofia 78, 95123 Catania, Italy; giuseppe.baratta@inaf.it (G.A.B.); maria.palumbo@inaf.it (M.E.P.); carlotta.scire@inaf.it (C.S.); 3 Institute of Polymers, Composites and Biomaterials, National Research Council (IPCB-CNR), Via P. Gaifami 18, 95126 Catania, Italy

**Keywords:** space materials, polymethyl methacrylate, ion beam irradiation, polymer degradation pathway, solar wind

## Abstract

Space exploration missions are currently becoming more frequent, due to the ambition for space colonization in sight of strengthening terrestrial technologies and extracting new raw materials and/or resources. In this field, the study of the materials’ behaviour when exposed to space conditions is fundamental for enabling the use of currently existing materials or the development of new materials suitable for application in extra-terrestrial environments. In particular, the versatility of polymers renders them suitable for advanced applications, but the effects of space radiation on these materials are not yet fully understood. Here, to shed light on the effects of simulated solar wind on a polymeric material, polymethyl methacrylate (PMMA) was produced through radical bulk polymerization. The PMMA in the form of a thin film was subjected to proton beam bombardment at different fluences and in a high vacuum environment, with structural changes monitored through real-time FT-IR analysis. The structure of the residual material was investigated through MALDI-TOF mass spectrometry and ^1^H-NMR spectroscopy. The collected data allowed us to hypothesize the structural modifications of the PMMA and the related mechanisms.

## 1. Introduction

Humankind’s ambition for space exploration is testified by the space agencies’ scientific efforts and expeditions since the 1960s. Recently, a new push has come from different space-exploration missions launched by major space agencies [1], such as NASA’s Artemis project which aims to bring astronauts back to the Moon’s surface in the coming years and ensure their settling on the satellite by 2030 [2]. To explore extra-terrestrial environments or to live in outer-terrestrial orbit (i.e., space stations), it is extremely important to use high-performance materials with suitable structural and chemical–physical characteristics. Indeed, these materials must be resistant to non-conventional environments with peculiar chemical and physical stresses (i.e., vacuum, ionizing radiations, thermal cycles, etc.), ensuring reliability and safety during their use [3,4,5]. This represents a key concept for structural materials, which must be easily realizable directly in situ, also exploiting local resources to overtake the need for commodity supply from the Earth [4]. However, considering the human crew onboard the spacecraft for mid-/long-term expeditions, the materials adopted for indoor applications are also of primal interest. Such an environment is not so far off. For instance, consider the International Space Station (ISS) which travels in the Low Earth Orbit (LEO) at about 300–400 km from sea level and is partially subjected to these space stresses.

The awareness of the operative conditions in outer orbit environments is fundamental to developing proper materials. Concerning the lunar surface and/or space, over vacuum and thermal cycles, the main problems are caused by different types of charged particle radiation sources: (i) Galactic Cosmic Rays (GCRs), a source of high-energy radiation mostly composed of atomic nuclei (mainly proton and helium ions, with energy ranging from a few MeV/nucleon to 10^4^ MeV/nucleon) and a smaller part of electrons and positrons; (ii) solar energetic particles (SEPs), composed of medium-energy particles emitted by the Sun during different types of solar events, mostly protons and alpha particles having energies of the order of MeV; (iii) solar wind particles with an energy of the order of 1 keV/u mainly composed of protons and alpha particles; (iv) electromagnetic radiation in a large range of energies (i.e., from gamma rays to microwaves) [6,7]. When high-energy particles collide with spacecraft materials, they can penetrate the spacecraft and emit secondary particles, which are dangerous to human health [8]. The braking processes and the consequent chemical effects induced by particles and radiation must be taken into account to develop materials suitable for applications in extra-terrestrial environments. The high-energy particles are currently braked with apposite protective materials exploiting the properties of metals and polymers [8], such as aluminium, polyethylene and polyimide-based composite materials [9,10,11]. 

Nevertheless, the polymers’ versatility makes them suitable for advanced applications in extra-terrestrial environments, despite the effects of space radiation which may cause different degradation processes in these materials [5,12,13]. Exposure to a high vacuum may cause outgassing effects consisting in the loss of low-molecular-weight fragments (such as plasticizers) and the desorption of adsorbed gases [14]. Instead, high energy particles and/or radiation cause a more severe effect on the polymer matrices, depending on the energy range of the particle/radiation impinging on the polymer matrix. The occurrence of main chain fragmentation, depolymerization or the loss of side-chain groups induces the lowering of the polymer molecular weight. Instead, crosslinking reactions between different chains can increase the molecular weight and enhance the brittleness [5,12,13,15,16]. However, each polymer matrix shows different behaviour in response to space stresses, and it is possible to distinguish different degradation pathways depending on the stress condition to which the polymer is subjected. As an example, in these conditions, aromatic polymers usually show higher stability than polymers with labile groups [17,18].

Polymethyl methacrylate (PMMA) is a thermoplastic polymer with excellent mechanical properties, widely used for different applications, such as structural applications and also for windows and optical parts, due to its optical properties, e.g., glass [19]. Moreover, PMMA is simple to synthesize and can be used to produce polymeric-based nanocomposites possessing different characteristics and applications based on the used nanofiller [20,21,22,23,24]. PMMA is already widely used in form of thin film for the production of different types of electrical and optical devices [25,26], the preparation of superhydrophobic and antireflective glass surfaces [27], as well as the preparation of protective coatings when mixed with inorganic fillers [28], thanks to the ability to tailor its properties depending on the synthesis method and the surface modifications [29].

Concerning ion beam bombardment experiments performed on PMMA, in general, irradiating the polymer matrix with both light or heavy ions, at high fluences, results in polymer backbone degradation [30] and the formation of amorphous hydrogenated carbon materials [31,32]. However, there are few studies about the degradation mechanisms that occur when PMMA is subjected to ion bombardment or electromagnetic radiation [33,34], without focusing on extra-terrestrial stresses. This work concerns a preliminary study on the behaviour of PMMA thin films when exposed to environmental conditions partially simulating the space conditions. 

The pristine PMMA was synthesized through radical bulk polymerization and its structure was investigated through FT-IR and NMR spectroscopies and MALDI-TOF mass spectrometry. To investigate the PMMA behaviour in a simulated solar wind environment, the PMMA thin films were subjected to high vacuum and H^+^ ion beam bombardment. In particular, the thin film was irradiated at different ion fluences, meanwhile monitoring the structural changes through real-time FT-IR analysis. Finally, the structure of the residual material was investigated through MALDI-TOF mass spectrometry and ^1^H-NMR spectroscopy. Based on the experimental results, the structural modifications occurring to the polymer matrix were discussed, hypothesizing the structure of the modified PMMA and the related mechanisms. To our knowledge, this is the first study in which MALDI-TOF mass spectrometry and NMR techniques have been used to study the structural modification of PMMA subjected to a simulated solar wind.

## 2. Materials and Methods

Methyl methacrylate (MMA) and 2,2′ azo-bis isobutyronitrile (AIBN) were purchased from Fluka. The Tetrahydrofuran (THF), *n*-hexane, dichloromethane, diethyl-ether and dimethyl sulfoxide (DMSO-d_6_) were purchased from Sigma Aldrich (Merk Life Science S.r.l., Milan, Italy).

### 2.1. Synthesis of Polymethylmethacrylate

PMMA was obtained through bulk radical polymerization of MMA using AIBN as the thermal initiator. Briefly, the inhibitor-free MMA (14 g, 140 mmol) was mixed with AIBN (116 mg, 0.7 mmol) in a vial and set in an ultrasonic bath for 30 min. Then, the mixture was placed under stirring in an oil bath (55 °C) for 20 h. The obtained polymer was solubilized in THF and precipitated in n-hexane. 

Thin films of PMMA were produced through a drop-casting procedure onto a water bath. An aliquot (130 μL) of a PMMA solution (dichloromethane/diethyl ether 50:50) was dropped onto the water bath. Then, the floating thin film was recovered and dried under the nitrogen flux for two hours and in a vacuum oven at 50 °C for 25 h.

### 2.2. Instruments

MALDI-TOF mass spectra were acquired with a Voyager DE (PerSeptive Biosystem, Perkin Elmer, Waltham, MA, USA), detecting positive ions in the linear-mode and using the delay extraction procedure (25 kV applied after 2600 ns, a potential gradient of 454 V mm^−1^ and a wire voltage of 25 V) [35,36]. The samples were prepared using Trans-2-[3-(4-tert-Butylphenyl)-2-methyl-2-propenylidene] malononitrile (DCTB) as a matrix. The mass spectrometer calibration was performed as previously described [37]. The molecular weights were determined through Grams software (PerSeptive Biosystem, Perkin Elmer, Waltham, MA, USA) [38] and the m/z value reported indicates the molecular ion, taking into account the most abundant isotope of each element in the molecule.

^1^H-NMR spectra were acquired using a ^UNITY^INOVA instrument (Varian, Agilent Technologies, Santa Clara, CA, USA) operating at 500 MHz (^1^H), setting the sample temperature at 27 °C. Spectra acquisition and processing were performed using VnmrJ software (Version 2.2C, Varian, Agilent Technologies, Santa Clara, CA, USA). The samples were dissolved in DMSO-d_6_ and the chemical shifts were expressed in ppm.

Gel Permeation Chromatography (GPC) experiments were performed using a PL-GPC 110 (Polymer Laboratories, Agilent Technologies Deutschland GmbH, Böblingen, Germany) thermostated system, equipped with two Mixed-D and one Mixed-E PL-gel 5 μm columns joined in series. The instrument is interfaced with a differential refractometer (DR) detector connected in parallel with a UV–Vis spectrophotometer (Hewlett Packard series 1050) and DAWN multi-angle laser light scattering (Wyatt Technology, Santa Barbara, CA, USA) detectors, connected in series. The analyses were performed at 35 ± 0.1 °C using tetrahydrofuran (THF) as an eluent at a flow rate of 1 mL/min. The dn/dc value for PMMA in THF was fixed at 0.085 mL/g [39]. The acquired data were analysed with ASTRA 6.0.1.10 software (Wyatt Technology, Santa Barbara, CA, USA).

The FT-IR spectra were processed using the Spectragryph optical spectroscopy software [40]. 

Ion irradiation experiments have been performed at Laboratorio di Astrofisica Sperimentale (LASp) of the Istituto Nazionale di Astrofisica (INAF)—Osservatorio Astrofisico di Catania. For this purpose, a Danfysik 1080 ion accelerator upgraded to an energy of 200 keV has been used. The effects induced by ion irradiation have been studied in situ, i.e., under vacuum at different irradiation fluences, by MIR spectroscopy by using a Bruker Vertex 70 spectrometer in the range (10,000–400 cm^−1^) working at a resolution of 1 cm^−1^ with a sampling of 0.25 cm^−1^. 

### 2.3. Ion Bombardment Experiments 

**Figure 1:**
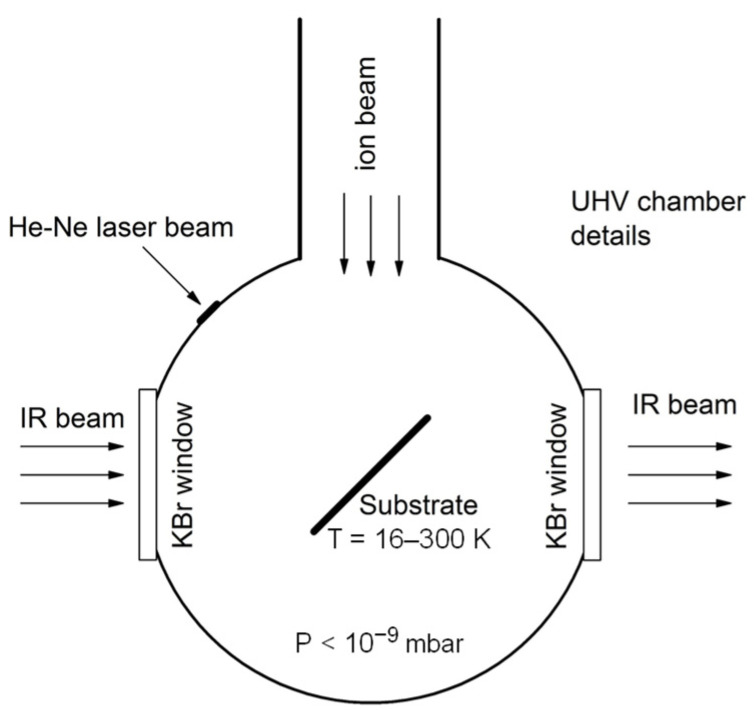
Simplified scheme of the apparatus for ion irradiation experiments.

The experimental set-up (Figure 1) consists of a stainless steel ultrahigh vacuum (UHV) chamber with a base pressure lower than 10^−9^ mbar [41]. Fast ions, up to an energy of 200 keV (400 keV for double ionization), are obtained by the 200 kV Danfysik implanter installed in a separate vacuum line (with a base pressure of ~1 × 10^−^^7^ mbar) that is connected to the UHV chamber through a UHV gate valve. In this work, the PMMA irradiation was carried out with 200 keV H^+^ ions. The proton-ion beam is focused along the beam-line and its spot is electrostatically swept in order to obtain uniform coverage of the irradiated sample. The ions fluence is recorded in situ by a current integrator. The Fourier-transform infrared (FTIR) spectrometer Vertex 70 is interfaced to the UHV chamber through two KBr IR transparent windows. The spectrometer is placed on a moveable optical bench that allows precise alignment of the IR beam with respect to the substrate. The substrate holder is inclined by 45 degrees with respect to both the ion beam and IR beam directions; hence IR transmission spectra of the irradiated samples can be acquired in situ, through a hole made in the sample holder, before, during, and after irradiations. The chemical effects induced by ion irradiation depends, in first approximation, on the total dose regardless of the ion mass and energy. The total dose is computed by multiplying the ion fluence (cm^−2^) for the stopping power (eV cm^2^ 16u^−1^) where 16u represents a small molecule.

The corresponding dose is given in eV/16u. The stopping power changes with the ion mass and energy, hence different ions with different energy give the same chemical effects at the same irradiation dose even if the fluence can be different if they have different stopping power. By increasing the angle of incidence of ions from zero to 45 degrees, the stopping power increases since the penetration depth of ions decreases. Hence, irradiation at normal incidence would give the same effects of irradiation at 45 degrees of incidence, that is our configuration, at a slightly higher fluence. By using the TRIM Monte Carlo simulation software [42] and our PMMA film parameters, we observed that normal incidence irradiation would give the same dose at a 22% higher fluence with respect to 45 degree irradiation for 200 keV H^+^. At any rate, it should be considered that in space, for sufficiently long periods of time, irradiation occurs from all directions.

## 3. Results

With the aim to better understand the behaviour of PMMA when subjected to proton bombardment, PMMA was synthesized by bulk radical polymerization (at 55 °C for 20 h) using AIBN as a thermal initiator. Finally, the purified PMMA (by precipitation in n-hexane from THF solution) was characterized by GPC, MALDI-TOF MS and ^1^H-NMR spectroscopy.

**Figure 2:**
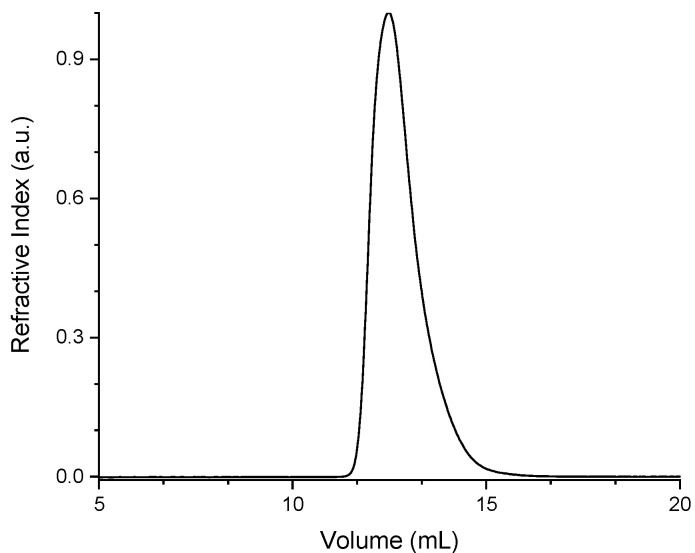
GPC, Refractive Index trace of PMMA.

The GPC analysis of PMMA (Figure 2) shows a quasi-Gaussian distribution, the calculated average molecular weights for PMMA are: 3.06 × 10^5^ Da for Mn and 6.29 × 10^5^ Da for Mw (PDI = 2.06). 

**Figure 3:**
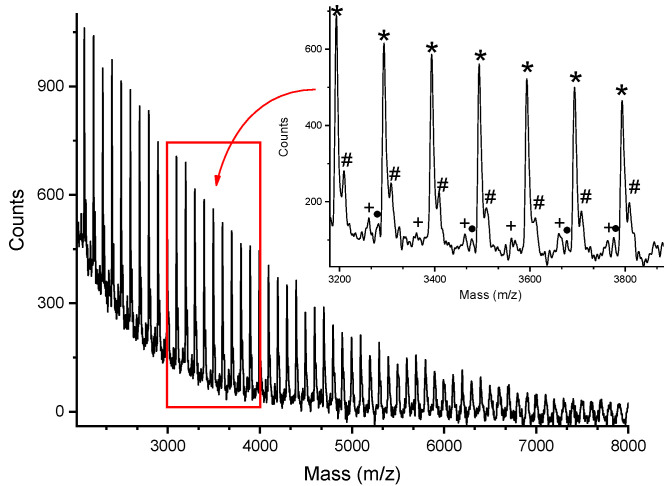
MALDI-TOF mass spectrum of pristine PMMA. The peaks are relative to chains terminated by disproportionation (“*”, MNa^+^; “**#**”, MK^+^) and by coupling mechanism ( “+”, MNa^+^; “•”, MK^+^).

The MALDI-TOF mass spectrum of the pristine PMMA (Figure 3) shows peaks due to both the termination by disproportionation (peaks at m/z = 69 + n × 100 + 23, “*”, detected as MNa^+^; peaks at m/z = 69 + n × 100 + 39 “**#**”, detected as MK^+^, with n values from 20 to 78) and by coupling mechanism (peaks at m/z = 68 × 2 + n × 100 + 23, “+”, detected as MNa^+^; peaks at m/z = 68 × 2 + n × 100 + 39, “•”, detected as MK^+^, with n values from 20 to 78). The peaks belonging to termination by the disproportionation phenomenon are assigned considering the average value of polymeric chains having both saturated and unsaturated end-groups. Considering the relative intensities of the peaks, the polymerization termination by disproportionation phenomenon was prevalent.

The ^1^H-NMR analyses of the pristine PMMA confirm the structure assigned by MALDI-TOF MS. In particular, the ^1^H-NMR spectrum showed the typical signals of PMMA [43] (500 MHz, DMSO-d_6_): 6.9–5.9 ppm (weak signals, due to the termination by disproportionation); 3.57 ppm (3H, methyl-ester protons); 2.9–2.7 ppm (weak signals, due to the termination by disproportionation); 2.18–1.31 ppm (2H, methylenic protons); 1.20–0.53 ppm (3H, α-methyl protons, isotactic, atactic and syndiotactic). Signals belonging to the isobutyronitrile end-groups are not visible since they are hidden by the signals of the PMMA protons in the range 1.5–1.2 ppm.

Among the factors that afflict materials facing the out-of-earth environment, the pressure variation was first approached.

**Figure 4:**
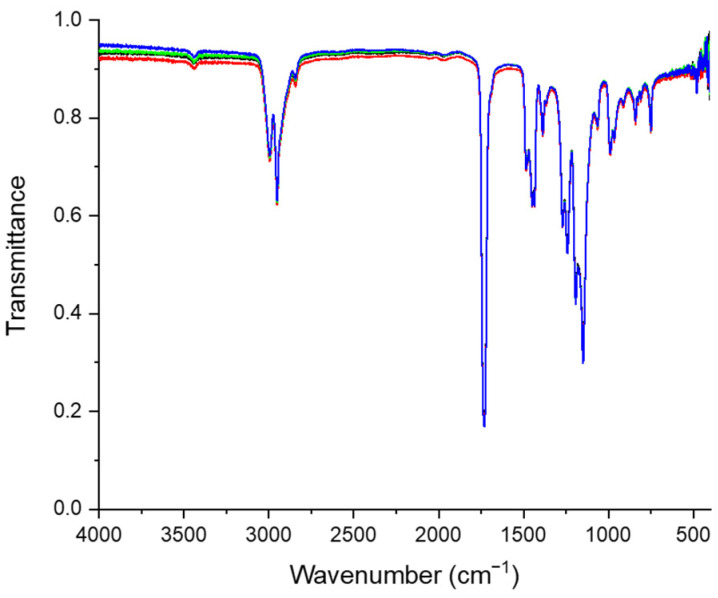
FT-IR spectra of a PMMA thin film at 10^−7^ mbar pressure and acquired after 0 (black line), 6 (red line), 25 (green line) and 30 (blue line) hours (room temperature).

To verify the vacuum stability of the polymeric structure, a thin film of PMMA was placed in the LASp vacuum chamber at room temperature (RT), monitoring the effect of a prolongated vacuum condition (10^−7^ mbar) through in situ FT-IR spectroscopy. As shown in Figure 4, there are no significant differences in the acquired spectra during 30 h of treatment. These data suggest that the chemical structure of the polymer is not significantly affected by high-vacuum conditions, confirming the negligible presence of any volatile compounds inside the polymeric material (residual from the production processes) and the absence of a significant amount of outgassing species.

**Figure 5:**
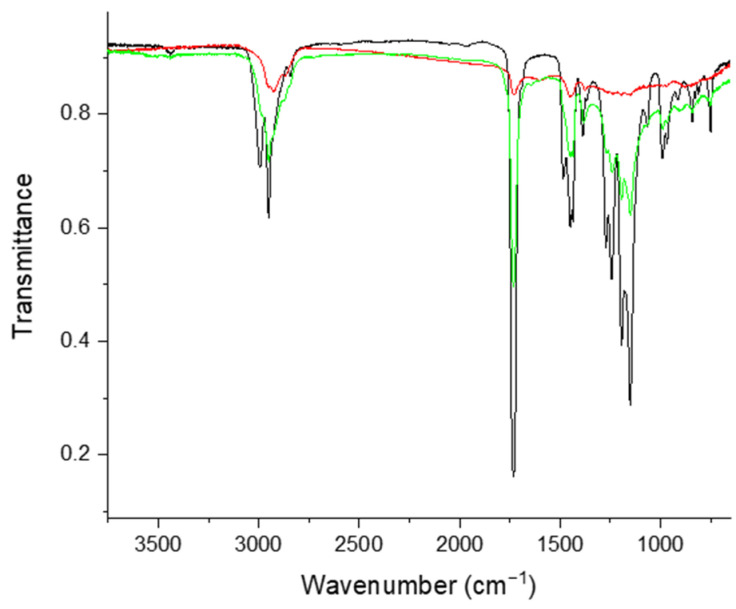
FT-IR spectra of PMMA thin film acquired during ion irradiation at different ion fluences: pristine PMMA (black line), after 1.3 × 10^15^ ions × cm^−2^ (green line) and after 9.6 × 10^15^ ions × cm^−2^ (red line) ion irradiation (200 keV H^+^).

The effects of a simulated solar wind on the polymer matrix were investigated by irradiating a thin film of PMMA with a 200 keV H^+^ ion beam at increasing ion fluences, monitoring the polymer structural variations through in situ FT-IR spectroscopy. The FT-IR spectra of the thin-film before ion irradiation (Figure 5, black line) show vibrational peaks typical of the PMMA matrix [44] as reported in Table 1.

The 200 keV proton irradiation steps were performed in the fluence range from 6.25 × 10^12^ ions × cm^−2^ to a total fluence of 9.6 × 10^15^ ions × cm^−2^. In Figure 5, the IR spectra of the pristine PMMA (black line), after 1.3 × 10^15^ ions × cm^−2^ (green line) and after 9.6 × 10^15^ ions × cm^−2^ (red line) are shown. The progressive intensity decrease of the PMMA signals at increasing ion fluences is evident. In particular, the variations of the signals attributed to -CH_3_ stretching (2995 and 2950 cm^−1^), -CH_2_ stretching (2840 cm^−1^) and -CH_3_ bending (1486 and 1386 cm^−1^) could be interpreted as a partial decomposition of the polymer chain, due to reactions inducing the loss of methyl and carboxy-methyl pendant groups. Indeed, the strong intensity decrease of the C=O stretching signal (1730 cm^−1^) suggests the occurrence of a de-carboxymethylation reaction, while the appearance of a signal at 1605 cm^−1^, attributable to the conjugated C=C stretching, could suggest the formation of unsaturated moieties (discussed in Section 4). Furthermore, the disappearance of all the signals belonging to C-O-C stretching further supports the de-carboxymethylation mechanism.

Moreover, the remaining signals in irradiated PMMA at 2950 cm^−1^ (CH_3_ asym. stretching), 2923 cm^−1^ (CH_2_ asym. stretching), 2870 cm^−1^ (CH_3_ stretching), 1451 cm^−1^ and 1376 cm^−1^ (CH_3_ bending) [45], could suggest the formation of a polypropylene-like structure, due to the loss of methyl-ester groups.

**Figure 6:**
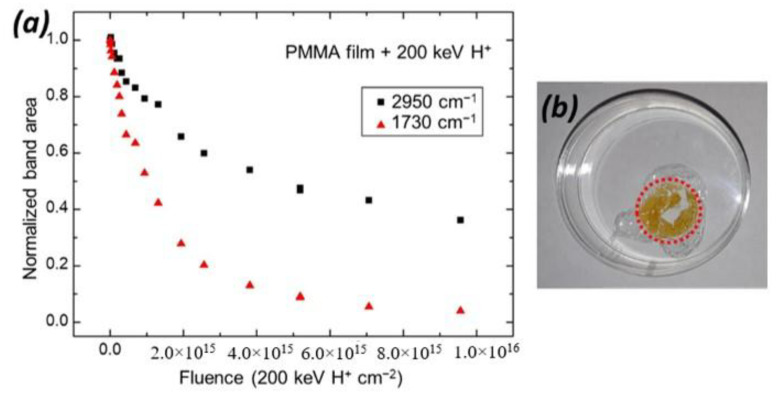
(**a**) Variation of the normalized band area of -CH stretching (underlying area of the peak at 2950 cm^−1^, black) and C=O stretching signal (underlying area of the peak at 1730 cm^−1^, red) as a function of ion fluence. (**b**) Picture of PMMA thin film after the ion-beam irradiation. The red circle indicates the bombarded area.

As a preliminary investigation of the magnitude of de-carboxymethylation and demethylation reactions, the related PMMA IR absorption features were integrated and normalized to their initial value. The normalized band area variations as a function of the ion fluences were reported for the signals belonging to CH groups (spectrum range from 3065 to 2800 cm^−1^, Figure 6a, black dots) and to the carbonyl of the methyl-ester moieties (1730 cm^−1^, Figure 6a, red dots). Since the methyl group is present both in the α position and in the methyl-ester group, the signal at 2950 cm^−1^ should decrease faster than the carbonyl groups. However, signals belonging to alkane moieties lie beneath the large band from 3065 to 2800 cm^−1^. Thus, the formation of new -CH, -CH_2_ and/or -CH_3_ moieties lowers the speed in the decreasing of the methyl signals.

After ion bombardment, the colourless PMMA film exhibited a strong colour change to yellow–brown (Figure 6b), restricted to the ion-treated area. The two sample regions were isolated, collected separately and solubilized in DMSO-d_6_. As a confirmation of the polymer chemical modifications from the ion treatment, the irradiated area was demonstrated as less soluble than the colourless counterpart, suggesting the occurrence of cross-linking reactions.

**Figure 7:**
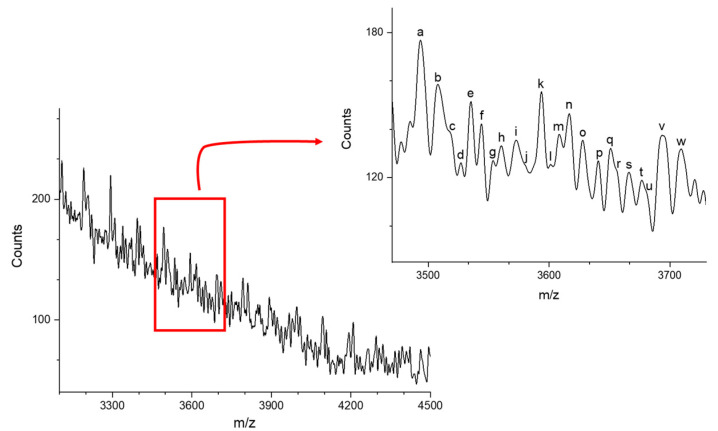
PMMA mass spectrum after bombardment with 200 keV H^+^ ion beam.

The effects of the impact of high-energy protons (simulating solar wind) on the chemical structure of PMMA were further investigated by analysing the soluble fraction of the yellow–brown area through MALDI-TOF mass spectrometry and ^1^H-NMR spectroscopy. The MALDI-TOF mass spectrum of the treated PMMA (Figure 7) shows a complex series of peaks (Table 2), afflicted by many isobars. It is possible to observe some more intense peaks belonging to both pristine PMMA (peaks at m/z = 69 + n × 100 + 23, with n values ranging from 31 to 41) which remained after the ion beam bombardment, and some different isobaric copolymeric species, described in detail in Table 2.

**Scheme 1:**
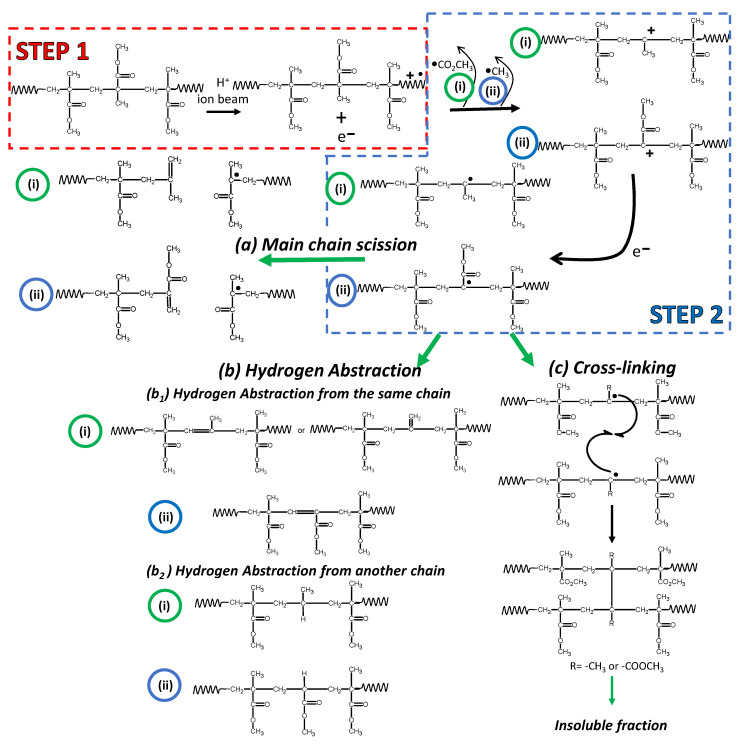
Schematic representation of the reactions occurring during the H^+^ ion-beam bombardment of PMMA.

The data analysis showed that the polymer structure alteration was due to the partial loss of side-groups, determining different repeating units within the polymer chain, as confirmed by the assignment of peaks to different copolymeric structures (as described in the Discussion section and represented in Scheme 1 routes b_1_ and b_2_, respectively). 

For brevity, Table 2 reports only the peaks related to the copolymeric structure containing MMA and the newly-formed saturated repetitive units (after the loss of α-methyl and/or methyl-ester groups). The copolymeric structure attributions are codified as N_(x,y,z)_, where: N = total (x + y + z) number of repeating units in the oligomeric species; x = number of MMA units; y = number of de-carboxymethylated units; z = number of de-methylated units. EG represents both -H and/or -IBN (isobutyronitrile) end-groups.

**Figure 8:**
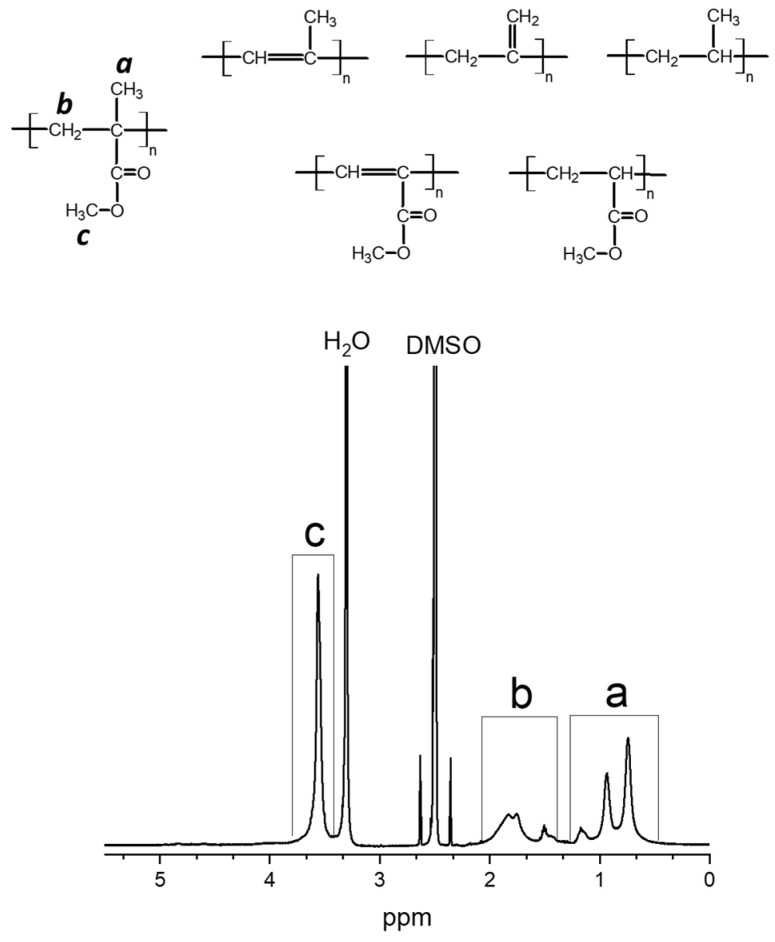
^1^H-NMR spectra of irradiated PMMA, with the possible repeating unit structures (top).

The ^1^H-NMR spectrum of the bombarded PMMA (Figure 8) shows the following signals (500 MHz, DMSO-d_6_, 27 °C): 3.57 ppm (c), 2.02–1.31 ppm (b), 1.18–0.65 ppm (a), belonging to the pristine PMMA. Peaks belonging to the new repeating units formed after the ion-beam bombardment are not evident in the ^1^H-NMR spectrum because the signals of the new repetitive units fall in the same region of the PMMA peaks. However, some extremely weak signals seem to appear in the chemical-shift range 8.2–7 ppm, suggesting a possible partial aromatization of segments of the PMMA.

Despite the results obtained by FT-IR, MALDI-TOF and ^1^H-NMR appearing to not be in accordance, the partial solubility of irradiated PMMA must be considered. Indeed, FT-IR analyses, performed on the entire thin film having linear, branched and cross-linked chains, provide an overview of all the functional groups and transformations incurred by the polymer matrix. On the contrary, the MALDI-TOF and ^1^H-NMR analyses provide information only about the soluble fraction. 

## 4. Discussion

The effects of simulated solar wind on PMMA thin films were investigated by different analytical techniques, highlighting the differences between pristine and irradiated samples. The data show that the ion-beam bombardment causes deep structural modifications in the polymer matrix, due to the loss of the side-chain groups, leading to a copolymer composed of different repeating units.

The experimental results allowed us to hypothesize two different degradation pathways occurring during the ions’ impact (Scheme 1). Firstly, the proton beam impinging on the polymeric thin film (Scheme 1, Step 1) probably generates a radical cation on the main chain, causing the ejection of an electron [33]. Then, the expulsion of methyl-ester radicals (path i) and/or the α-methyl radicals (path ii) can occur, leaving a tertiary carbocation on the main chain which can be converted into a radical by capturing an electron (Scheme 1, Step 2) [46]. At this point, the polymeric chain can undergo two different reactions: (a) The presence of the radical could lead to the main chain scission, causing a lowering of the molecular weight of the polymer; (b) the polymeric chain could be stabilized by hydrogen abstraction from the same chain (route b_1_), creating double bonds, or from a different polymeric chain (route b_2_), producing a saturated chain and creating another propagating radical; (c) if two radicals are close enough, they could collapse leading to branching. This last occurrence was proven by MALDI-TOF analysis, which showed peaks belonging to polymeric structures containing more than three end-groups. The eventual recurrence of this mechanism could lead to the crosslinking of the polymer matrix, justifying the reduced solubility of irradiated PMMA than that of pristine polymer.

## 5. Conclusions

In this work, a thin film of PMMA was produced and subjected to 200 keV proton-beam bombardment to study its behaviour when exposed to simulated solar wind. The online FT-IR spectroscopy showed the disappearance of side-chain groups with different loss rates, and the formation of a new saturated copolymeric backbone. These data were confirmed by MALDI-TOF mass spectrometry that showed a lowering of the amount of the homopolymer peaks and the appearance of new peaks attributable to a copolymer-like composition. ^1^H-NMR provided noticeably different results from the above-discussed techniques, due to the weakness of the copolymer signals and their concealment behind the PMMA residual signals. Finally, the PMMA film solubility variation following ion irradiation represents a further confirmation of the branching/crosslinking reactions occurring between the polymeric chains. 

## Figures and Tables

**Table 1:** Assignment of FT-IR bands in pristine PMMA.

Wavenumber (cm^−1^)	Assignment
2995	α-CH_3_ asym. stretching; OCH_3_ asym. stretching
2950	α-CH_3_ sym. stretching; OCH_3_ sym. stretching; CH_2_ stretching
2840	CH_2_ stretching
1730	C=O stretching
1486	α-CH_3_ asym. bending
1450	CH_2_ bending
1436	O-CH_3_ sym. bending
1386	α-CH_3_ sym. bending
1270–1240	C-C-O stretching
1190–1150	C-O-C stretching
1062	C-C skeletal stretching
988	O-CH_3_ rocking
840	CH_2_ rocking
750	C-C skeletal stretching

**Table 2:** Table reassuming the repeating units’ composition of the bombarded PMMA.

Peak	[M]Na^+^ (m/z)	N_(x,y,z)_ = 36	N_(x,y,z)_ = 37	N_(x,y,z)_ = 38	N_(x,y,z)_ = 39
a	3493	M_28,2,6_ EG = H, IBN; * (M_34,0,0_ EG = H, IBN)	M_33,4,0_ EG = H, H	M_30,5,3_ EG = H, H	M_27,6,6_ EG = H, H
b	3507	M_29,2,5_ EG = H, IBN		M_31,5,2_ EG = H, H	M_28,6,5_ EG = H, H
c	3517	M_28,3,5_EG = IBN, IBN		M_30,6,2_ EG = H, IBN	
d	3527	M_32,1,3_ EG = H, H	M_29,2,6_ EG = H, H		
e	3535	M_31,2,3_ EG = H, IBN	M_28,3,6_ EG = H, IBN	M_33,5,0_ EG = H, H	M_30,6,3_ EG = H, H
f	3544	M_30,3,3_ EG = IBN, IBN	M_30,5,2_ EG = IBN, IBN; M_27,4,6_ EG = IBN, IBN	M_27,6,5_ EG = IBN, IBN; M_32,6,0_ EG = H, IBN	
g	3553	M_34,1,1_, EG = H, H	M_34,3,0_ EG = H, H; M_31,2,4_ EG = H, H	M_31,4,3_ EG = H, H	M_28,5,6_ EG = H, H
h	3561	M_31,3,2_ EG = IBN, IBN; M_28,2,6_ EG = IBN, IBN	M_33,4,0_ EG = H, IBN	M_30,5,3_ EG = H, IBN	M_27,6,6_ EG = H, IBN
i	3573	M_32,3,1_ EG = IBN, IBN	M_29,4,4_ EG = IBN, IBN		
j	3580	M_31,1,4_ EG = H, IBN		M_33,4,1_ EG = H, H	M_30,5,4_ EG = H, H
k	3593	M_32,1_,_3_ EG = H, IBN; * (M_35,0,0_ EG = H, IBN)	M_29,2,6_ EG = H, IBN	M_34,4,0_ EG = H, H	M_31,5,3_ EG = H, H
l	3601		M_31,4,2_ EG = IBN, IBN	M_28,5,5_ EG = IBN, IBN	
m	3608	M_33,1,2_ EG = H, IBN; M_30,0,6_ EG = H, IBN	M_30,2,5_ EG = H, IBN		M_32,5,2_ EG = H, H
n	3617	M_32,2,2_ EG = IBN, IBN	M_29,3,5_ EG = IBN, IBN		M_31,6,2_ EG = H, IBN
o	3628		M_33,4,0_ EG = IBN, IBN	M_30,5,3_ EG = IBN, IBN	M_27,6,6_ EG = IBN, IBN
p	3641		M_34,1,2_ EG = H, H; M_31,0,6_ EG = H, H	M_31,2,5_ EG = H, H	
q	3651	M_33,0,3_ EG = H, IBN	M_30,1,6_ EG = H, IBN	M_35,3,0_ EG = H, H	M_32,4,3_ EG = H, H
r	3656		M_32,0,5_ EG = H, H	M_32,5,1_ EG = IBN, IBN	M_29,6,4_ EG = IBN, IBN
s	3666	M_34,0,2_ EG = H, IBN	M_31,1,5_ EG = H, IBN		M_33,4,2_ EG = H, H
t	3677	M_30,0,6_ EG = IBN, IBN	M_35,2,0_ EG = H, IBN	M_32,3,3_ EG = H, IBN	M_29,4,6_ EG = H, IBN
u	3680	M_35,0,1_ EG = H, IBN	M_32,1,4_ EG = H, IBN		M_34,4,1_ EG = H, H
v	3693	M_36,0,0_ EG = H, IBN	M_33,1,3_ EG = H, IBN	M_30,2,6_ EG = H, IBN	M_35,4,0_ EG = H, H
w	3709		M_31,0,6_ EG = H, IBN	M_36,2,0_ EG = H, H	M_33,3,3_ EG = H, H

N**_(x,y,z)_**: N = total number (x + y + z) of repeating units inside the oligomeric species; x = number of MMA units; y = number of de-carboxymethylated units; z = number of de-methylated units. EG represents both -H and/or -IBN (isobutyronitrile) end-groups. * Species in brackets are related to pristine PMMA.

## Data Availability

Not applicable.
